# Retrospective Drug Utilization Pattern of the Use of Antivirals and Its Outcome in Hospitalized COVID-19 Patients in a Tertiary Care Hospital in Mumbai From July to October 2020

**DOI:** 10.7759/cureus.108478

**Published:** 2026-05-08

**Authors:** Krisha R Marolia, Rajan P Nerurkar

**Affiliations:** 1 Pharmacology, Topiwala National Medical College & B. Y. L. Nair Charitable Hospital, Mumbai, IND

**Keywords:** clinical improvement, ivermectin, regression analysis, remdesivir, sars-cov-2

## Abstract

Background

The initial recommended treatment at the start of the COVID-19 pandemic in India included isolation, symptomatic treatment, oxygen support, empirical antibiotics, and hydroxychloroquine prophylaxis. However, evolving guidelines and limited evidence on antiviral efficacy highlighted a gap in evidence-based treatment approaches. Recognizing this gap, a study was planned to assess the antiviral impact at our institute. We aimed to study the prescription pattern of antivirals in hospitalized COVID-19 patients over four months and analyse the influence of age, gender, antiviral use, comorbidities, and oxygen requirement on outcomes: clinical improvement and hospital stay duration.

Methods

This retrospective observational single-centre study included reverse transcription-polymerase chain reaction (RT-PCR)-confirmed COVID-19 patients who were hospitalized and received remdesivir, favipiravir, ivermectin, or oseltamivir. Descriptive statistics were analysed using Microsoft Excel 365 (Microsoft® Corp., Redmond, WA). Multiple linear regression and logistic regression models were used with JASP 0.16.3 software.

Results

Among 400 patient prescriptions, 5,172 drugs were recorded: 542 repurposed antivirals and 4,630 concomitant drugs. Ivermectin (376, 69.37%) was the most frequently prescribed antiviral, followed by remdesivir (97, 17.9%), favipiravir (59, 10.89%), and oseltamivir (10, 1.85%). Nutritional supplements (1536, 33.1%) were the most common concomitant drug class, with vitamin C being the most prescribed. Logistic regression showed that male gender and oxygen therapy were positively associated with clinical improvement. Linear regression revealed that older age and higher disease severity correlated with longer hospital stays. Antiviral use showed no significant association with either disease improvement or duration of stay.

Conclusion

Ivermectin and remdesivir were the two most frequently used repurposed antivirals. While male gender and oxygen therapy were linked with clinical improvement, older age and severe disease predicted longer hospitalization. Antivirals themselves did not significantly affect outcomes, underlining the complexity of COVID-19 management.

## Introduction

Severe acute respiratory syndrome coronavirus-2 (SARS-CoV-2) was first identified in a seafood market in Wuhan, China, in December 2019 and was declared a pandemic by the WHO on March 11, 2020 [1]. As of November 14, 2022, the virus had infected approximately 631.9 million people worldwide, with 6.58 million fatalities [2]. SARS-CoV-2, an enveloped RNA beta coronavirus, spreads through inhalation or contact with infected droplets [3], causing disease severity ranging from asymptomatic [4] to critical, influenced by the host immune response [5].

In India, the Ministry of Health and Family Welfare (MoHFW) initially recommended isolation, symptomatic treatment, oxygen therapy, empiric antibiotics, and hydroxychloroquine prophylaxis [6]. As the pandemic evolved, the urgent need for effective antivirals led to multiple trials, including WHO's SOLIDARITY, DisCoVeRy [7], and RECOVERY trials [8], which evaluated the following drug arms: remdesivir, hydroxychloroquine, lopinavir/ritonavir, azithromycin, tocilizumab, and interferon-β1a [7,8]. The Inflammatory Bowel Disease and Recurrent Clostridium difficile Infection: Outcomes After Fecal Microbiota Transplantation (ICON) study in the USA assessed ivermectin [9]. The MoHFW later updated the guidelines in India to include anticoagulants, methylprednisolone, and investigational therapies such as convalescent plasma [1].

As knowledge of COVID-19 expanded, treatment strategies included repurposed and newly developed drugs. Due to limited evidence on the antiviral efficacy of these therapies, further evaluation was necessary. Recognizing this gap, a study was planned to assess antiviral impact at our tertiary care hospital, which was designated as a dedicated COVID hospital (DCH).

## Materials and methods

Objectives

The following are the objectives of the study: (1) to investigate the prescription pattern of antiviral drugs in COVID-19 patients over a period of four months in a tertiary care hospital; (2) to analyse the effect of independent parameters, such as age, gender, antiviral administration, comorbidity, and oxygen requirement, on clinical improvement of disease; and (3) to analyse the effect of independent parameters, such as age, gender, antiviral administration, comorbidity, oxygen requirement, and disease severity, on duration of hospital stay.

Methodology

We conducted a retrospective observational single-centre study in the medical records section of our tertiary care hospital and retrieved data from case paper records of COVID-19 patients admitted to our hospital. Simple random sampling was done by using a random number generation computer programme. The programme had sets of 1-100, and 7 random numbers were generated out of each set.

During the study period from July to October 2020, a total of 5,670 COVID-19 patients were admitted to our institute. According to the sample size calculator, for this 5,670 population with a 95% confidence level and 5% confidence interval, the sample size needed was 360, which was rounded off to 400 (this 400 is equal to 7% of the total population of 5,670). Since the number of admissions was inconsistent in each month, to have an even distribution, we have taken 7% of indoor patients from each month, as shown in Table 1.

**Table 1:** Number of admissions in each month and the sample selected from each month

Month/Year	No. of admissions	Sample size
July 2020	1728	122
August 2020	1108	78
September 2020	1452	102
October 2020	1382	98
TOTAL	5670	400

All hospitalised patients admitted from July to October 2020 in medical wards with laboratory confirmed SARS-CoV-2 infection, as confirmed by reverse transcription-polymerase chain reaction (RT-PCR), who had received any one of the following drugs repurposed as antivirals after admission - remdesivir, favipiravir, ivermectin, or oseltamivir - were included in the study. The following patients were excluded from the study: patients who had received antivirals before admission; those with acute respiratory distress syndrome (ARDS) or sepsis defined according to WHO staging of COVID-19 disease for disease severity [10]; those with severity grade 6 on the 7-point ordinal scale on the WHO Master Protocol (V.3.0, 3 March 2020) [7]; those with terminal stage cancer/malignancy; pregnant patients; and COVID-19 positive patients undergoing surgery.

The study period was from July 2020 to October 2020, and data were collected from the medical records office (MRO) between February 2022 and July 2022.

The case record form (CRF) captured the following parameters for assessment: demographics, presenting clinical features, associated comorbid condition(s), disease severity as per WHO staging (Table 2) and 7-point ordinal scale (Table 3), oxygen‑therapy requirements, and hospital‑stay duration.

**Table 2:** WHO staging of COVID-19 disease for disease severity SpO₂: Peripheral oxygen saturation; RR: Respiratory rate; ARDS: Acute respiratory distress syndrome; PaO₂/FiO₂: Partial pressure of arterial oxygen to fraction of inspired oxygen; PEEP: Positive end-expiratory pressure; CPAP: Continuous positive airway pressure Source: [10]

Disease Severity	Clinical symptoms	O_2_ Requirement
Asymptomatic	None	No
Mild	Fever, cough, sore throat, body ache, headache, Mild nasal congestion, anosmia, ageusia, diarrhoea	No evidence of hypoxia, SpO2 within normal range
Moderate	Pneumonia, but no signs of severe pneumonia, including SpO2 ≥ 90% on room air	SpO_2_ ≥90% and RR >24/min
Severe	Signs and symptoms of pneumonia	SpO_2_ <90% and RR>30/min
Critical	ARDS	Mild ARDS: 200 mmHg < PaO2/FiO2 a ≤ 300 mmHg (with PEEP or CPAP ≥ 5 cmH2O). Moderate ARDS: 100 mmHg < PaO2/FiO2 ≤ 200 mmHg (with PEEP ≥ 5 cmH2O). Severe ARDS: PaO2/FiO2 ≤ 100 mmHg (with PEEP ≥ 5 cmH2O)
Critical	Sepsis, septic shock, multi-organ failure	Acute life-threatening organ dysfunction

**Table 3:** Disease severity on the seven-point ordinal scale of the WHO Master Protocol (V.3.0, March 3, 2020), based on hospitalisation status and oxygen requirement ECMO: Extracorporeal membrane oxygenation Source: [7]

Score	Hospitalisation status	Activity/Oxygen requirement
1	Not hospitalised	No limitation on activities
2	Not hospitalised	Limitation on activities
3	Hospitalised	No supplemental oxygen required
4	Hospitalised	Supplemental oxygen required
5	Hospitalised	Supplemental oxygen required via non-invasive ventilation or high flow oxygen devices
6	Hospitalised	Supplemental oxygen required via invasive mechanical ventilation or ECMO
7	Death	

Prescription details recorded included antiviral drug therapy details such as route of administration, dose, frequency of administration, duration of therapy, and number of antivirals prescribed per prescription; concomitant medications were given - nutritional supplements, steroids, anticoagulants, antibiotics, and tocilizumab. The outcome details recorded were the effect of independent parameters on the improvement of the disease and the duration of hospital stay.

Plan of analysis: Descriptive statistics, such as mean, standard deviation, median, range, and percentage, were used to describe the data that were collected. Software used was Microsoft Excel 365 (Microsoft® Corp., Redmond, WA). Logistic regression and linear regression models were used to assess the effect of independent parameters on the improvement of the disease and the duration of stay, respectively. Software used was JASP 0.16.3 (University of Amsterdam, Netherlands).

## Results

In this study, the mean ± SD age of participants was 55.3 ± 15.33 years, with a range of 19-94 years. The study population showed a male preponderance (266, 66.5%). A total of 35 symptoms were seen at the presentation of the patient to the hospital. Most patients (135, 33.75%) presented with two symptoms to the hospital, of which fever and breathlessness were the most common, and nine (2.25%) patients were asymptomatic. Associated comorbid conditions were seen in 265 patients (66.25%), the majority of whom had hypertension and diabetes mellitus type 2.

The disease severity of the patients was assessed on admission, at day one, and at day seven of antiviral drug administration. Severity on admission was assessed using two parameters - WHO staging of COVID-19 disease (11) and WHO 7-point ordinal scale (7), which for the majority of the study population (224, 56%) was moderate and score 4, respectively. Disease severity on day one and day seven is summarised in Table 4.

**Table 4:** Disease severity on day one and day seven of antiviral administration according to the WHO ordinal scale and COVID disease staging (n = 542)

Disease severity		Frequency	
WHO 7-point ordinal scale score	WHO staging of COVID-19 disease	On Day 1	On Day 7
3	Asymptomatic	9 (1.66%)	8 (1.48%)
3	Mild	191 (35.24%)	286 (52.77%)
4	Moderate	328 (60.52%)	241 (44.46%)
5	Severe	14 (2.58%)	7 (1.29%)

The mean duration of hospital stay observed was 15.24 ± 6.03 days, with a range of 7-46 days. In our study, a total of 5,172 drugs were prescribed to the 400 patients. Of which, 542 were antivirals, and 4,630 were concomitant drugs. The average number of drugs prescribed per prescription was 12.93, of which the average number of antiviral drugs was 1.36, and the concomitant drugs prescribed per prescription were 11.57.

The most frequently prescribed antiviral agent was ivermectin (376, 69.37%), followed by remdesivir (97, 17.9%), favipiravir (59, 10.89%), and oseltamivir (10, 1.85%). The majority of the patients received one antiviral (266, 66.5%), two antivirals were given to 126 (31.5%) patients, and three antivirals were given to eight (2%) of the study population. In patients receiving two antivirals, ivermectin + remdesivir were the most common drugs given to 73 (18.25%) patients, followed by ivermectin + favipiravir, which were given to 47 (11.75%) patients. Table 5 summarizes the details of antiviral drug therapy.

**Table 5:** Antiviral drugs prescribed with their dosage and duration of therapy

Antiviral drugs	Dose	Frequency	Average duration of therapy in days	Range of duration of therapy in days
Tab. Ivermectin	12 mg	Once daily	2.9	1-10
Inj. Remdesivir	200 mg Loading dose on day 1, 100 mg from day 2	Once daily	5.08	1-10
Tab. Favipiravir	1800 mg loading dose on day 1, 800 mg from day 2	Twice daily	8.63	2-14
Tab. Oseltamivir	150mg	Once daily	7.89	1-13

The most common route of administration for antivirals was oral (445, 82.1%), followed by injectable (IV) (97, 17.9%). Out of concomitant drugs, nutritional supplements (1,536, 33.1%) were the most common class, of which vitamin C was the most prescribed. Antimicrobials, corticosteroids, and anticoagulants were the three classes of concomitant drugs that followed nutritional supplements, as seen in Table 6.

**Table 6:** Class of all concomitant drugs prescribed to the study population (n = 4,630)

Class of concomitant drugs	Frequency
Nutritional supplements	1536 (33.17%)
Antimicrobials	908 (19.61%)
Corticosteroids	411 (8.87%)
Anticoagulants	387 (8.36%)
Proton pump inhibitors	337 (7.3%)
Anti-hypertensives and anti-anginal drugs	291 (6.28%)
Anti-diabetic drugs	260 (5.61%)
Anti-platelet and lipid lowering agents	231 (5%)
Bronchodilators, cough expectorants and suppressants	63 (1.36%)
Anti-emetics, anti-diarrhoeal drugs and laxatives	60 (1.3%)
Miscellaneous drugs	38 (0.82%)
Anti-pyretic and anti-inflammatory drugs	33 (0.71%)
Thyroxine	21 (0.45%)
Anti-depressants, anti-psychotics, and sedatives	20 (0.43%)
Anti-histaminic drugs	19 (0.41%)
Biological agents	15 (0.32%)

A logistic regression model was used to assess the effect of age, gender, comorbidities, use of antivirals, and oxygen therapy, which were the independent variables, on improvement of disease severity, which was the dependent variable. We found that male gender and patients requiring oxygen were favourable factors for improvement of disease severity (Table 7).

**Table 7:** Improvement of disease severity by the multiple logistic regression model (p < 0.05) p < 0.05*, p < 0.001**

Independent variable	Odds ratio	95% Confidence interval (odds ratio scale)		p value
		Lower CI	Upper CI	
Age	0.995	0.974	1.016	0.629
Gender (male)	1.872	1.034	3.388	0.038*
Comorbidity (1)	0.857	0.414	1.774	0.678
Comorbidity (2)	0.935	0.411	2.128	0.874
Comorbidity (≥3)	1.588	0.614	4.102	0.34
Anti-viral (≥2)	1.682	0.914	3.098	0.095
Oxygen therapy (Yes)	69.389	31.303	153.817	<0.001**

A multiple linear regression model was used to assess the effect of age, gender, comorbidity, antiviral use, oxygen therapy, and disease severity as independent variables on the duration of hospital stay, which was the dependent variable. Older age and moderate-to-severe disease severity significantly increased hospital stay duration (Table 8).

**Table 8:** Duration of hospital stay by the multiple linear regression model (p < 0.05) p < 0.01*, p < 0.05**

Independent variable	Unstandardised coefficient	95% Confidence interval		p value
		Lower CI	Upper CI	
Age	0.056	0.015	0.098	0.008*
Gender (male)	0.091	-1.116	1.299	0.882
Comorbidity (1)	-0.131	-1.635	1.374	0.865
Comorbidity (2)	0.762	-0.878	2.401	0.361
Comorbidity (3)	-0.43	-2.378	1.519	0.665
Anti-viral (2)	0.823	-0.414	2.06	0.191
Oxygen therapy (Yes)	1.59	-0.235	3.415	0.087
Disease severity (moderate to severe)	1.827	0.031	3.623	0.046**

## Discussion

We studied the age distribution of the study subjects and found that the average age of the study population was 55.3 ± 15.33 years, which is similar to the study by Gokhale et al. [11]. As the age progresses, the age-related decrease in immune cell function and increased production of inflammatory cytokines may play a role in more admissions in the age group of 51-60 years [12].

Ivermectin was the most used antiviral (376, 69.37%), showing in vitro activity against SARS-CoV-2 [13,14] and cost-effectiveness over hydroxychloroquine and azithromycin [9,15]. The ICON trial reported lower mortality in ivermectin-treated patients on oxygen [9]. Chowdhury et al. found ivermectin-doxycycline superior in mild-to-moderate COVID-19, with faster RT-PCR negativity and symptom resolution [16]. Mahmud et al. concluded ivermectin-doxycycline reduced disease progression and improved recovery [17]. Remdesivir (97, 17.9%) was the second most used drug, recommended for moderate cases under Emergency Use Authorizations (EUA), except in severe renal impairment, liver dysfunction, pregnancy, or children [1]. ACTT-1 showed remdesivir shortened recovery time in hospitalized COVID-19 patients with lower respiratory tract infections [18]. Favipiravir was given to 59 patients (10.89%), with Zhao et al. reporting faster viral clearance and shorter RT-PCR negativity [19]. Oseltamivir (10, 1.85%) was administered twice daily (200 mg for five days); Chiba suggested its early use reduces fever in non-hypoxic COVID-19 outpatients [20].

Vitamin C, a potent antioxidant, enhances immune response and may prevent complications. It also restores endothelial function, potentially reducing lung inflammation and injury [21,22]. Corticosteroids (411, 8.87%) were used per the MoHFW guidelines for COVID-19 patients with rising oxygen needs or inflammation [1]. Methylprednisolone was shown to benefit older patients [23], those requiring oxygen, patients with raised inflammatory markers, and patients with severe disease or ARDS [24], but their use requires careful risk-benefit assessment.

## Conclusions

To conclude, we analysed 400 COVID-19 patient prescriptions in our study, documenting a total of 542 repurposed antiviral drugs. The most used antiviral was ivermectin, followed by remdesivir. Among concomitant medications, vitamin C, antimicrobials, corticosteroids, and anticoagulants were the most frequently administered classes. Improvement in COVID-19 severity was observed by day seven after antiviral initiation, particularly in male patients and those who received supplemental oxygen, as demonstrated by multiple logistic regression (adjusting for comorbidities, antiviral use, and age). Older individuals and those with moderate-to-severe disease had significantly longer hospital stays when analysed with linear regression, alongside other independent parameters, such as gender, comorbidities, antiviral use, and oxygen therapy. Antiviral therapy did not have a significant clinical impact either on disease improvement or on hospital stay duration in these models.
